# The effect of repeated remote ischemic postconditioning on infarct size in patients with an ischemic stroke (REPOST): study protocol for a randomized clinical trial

**DOI:** 10.1186/s13063-019-3264-0

**Published:** 2019-03-15

**Authors:** Thijs Landman, Yvonne Schoon, Michiel Warlé, Frank-Erik De Leeuw, Dick Thijssen

**Affiliations:** 1 Department of Physiology, Radboud University Medical Centre, Radboud Institute for Health Sciences, Geert Grooteplein Zuid 10, 6525GA Nijmegen, Gelderland Netherlands; 2 Department of Geriatric Medicine, Radboud University Medical Centre, Radboud Institute for Health Sciences, Geert Grooteplein Zuid 10, 6525GA Nijmegen, Gelderland Netherlands; 3 Department of Surgery, Radboud University Medical Centre, Geert Grooteplein Zuid 10, 6525GA Nijmegen, Gelderland Netherlands; 4 Centre for Cognitive Neuroscience, Department of Neurology, Radboud University Medical Centre, Donders Institute for Brain, Cognition and Behaviour, Geert Grooteplein Zuid 10, 6525GA Nijmegen, Gelderland Netherlands

**Keywords:** Stroke, Ischemia, Ischemic conditioning, Infarct size, Clinical outcome

## Abstract

**Background:**

Remote ischemic postconditioning (rIPostC) refers to the observation that repeated, short periods of ischemia protect remote areas against tissue damage during and after prolonged ischemia. Based on previous observations of a potential neuroprotective effect of rIPostC, the aim of this study is to evaluate whether repeated rIPostC after an ischemic stroke can reduce infarct size, which could be translated to an improvement in clinical outcomes.

**Methods/design:**

We will enroll 200 ischemic stroke patients to daily rIPostC or sham conditioning during hospitalization into a randomized single-blind placebo-controlled trial. The intervention consists of twice daily exposure to four cycles of 5-min cuff inflation around the upper arm to > 20 mmHg above systolic blood pressure (i.e., rIPostC) or 50 mmHg (i.e., control), followed by 5 minutes of deflation. The primary outcome is infarct size, measured using an MRI diffusion-weighted image at the end of hospitalization. Secondary outcomes include the Modified Rankin Scale, National Institutes of Health Stroke Scale, quality of life, and cardiovascular and cerebrovascular morbidity and mortality. To explore possible underlying mechanisms of rIPostC, venous blood will be sampled to assess biomarkers of inflammation and vascular health.

**Discussion:**

Previous studies in animals and humans, using a single bout of remote ischemic conditioning, report a potential effect of rIPostC in attenuating neural damage. Although repeated rIPostC has been investigated for cardiovascular disease patients and preclinical stroke models, no previous study has explored the potential physiological and clinical effects of repeatedly applying rIPostC during the hospitalization phase after a stroke.

**Trial registration:**

Netherlands Trial Register, NTR6880. Registered on 8 December 2017.

**Electronic supplementary material:**

The online version of this article (10.1186/s13063-019-3264-0) contains supplementary material, which is available to authorized users.

## Background

Ischemic stroke is the leading cause of disability in adults worldwide and has the second highest mortality of all cardiovascular diseases [1]. In particular, the intravenous and endovascular treatment of ischemic stroke has markedly improved survival and long-term functional outcomes [2–4]. Unfortunately, a substantial number of stroke patients still end up with a physical disability. This may in part be because the therapeutic window to attenuate the detrimental impact of ischemic injury is limited to 6 hours after the stroke. Currently, there are no subsequent treatment options available [5], although some recent studies have had promising results for intra-arterial therapy in specific subgroups of stroke patients [6, 7]. Moreover, acute revascularization might induce ischemia reperfusion injury [8] and glutamatergic excitotoxicity [9], which could lead to additional damage in the ischemic penumbra. This highlights the need for innovative additional treatment options, which preferably would extend beyond the 6 hours post-stroke. Identification of such treatment options may have a significant impact on the burden of stroke for individual patients, their caregivers, health-care professionals, and at the socioeconomic level.

A potential approach may be the application of remote ischemic postconditioning (rIPostC). Remote ischemic conditioning (RIC) consists of several cycles of brief periods (5 min) of limb ischemia followed by reperfusion, which can be applied by inflating a simple blood pressure cuff. This intervention subsequently confers protection against severe ischemia in remote organs in humans [10–12]. Due to the unpredictability of stroke, applying RIC before the event is not possible. However, similar protective effects are present when RIC is applied during or even after an ischemic insult [13], which is known as rIPostC. Whilst the majority of studies have focused on the protective effects of ischemic pre- and postconditioning on cardiac tissue [10–12, 14, 15], recently, several studies have supported the ability of rIPostC to reduce neural damage after reperfusion [8, 16–19], validating that rIPostC may have clinical potential for stroke patients, with clinical trials showing promising results [20, 21]. Additionally, Hess et al. postulated that, in addition to the short-lasting benefits of a single bout of conditioning, chronic benefits may be induced with repeated daily conditioning [18]. This has since been supported by preclinical studies in mice and rats [22, 23] and further supported by evidence from clinical trials in humans with favorable clinical outcomes for repeated rIPostC in ischemic stroke patients [24, 25].

We, therefore, hypothesized that rIPostC during the first few days following an ischemic stroke reduces infarct size. Since infarct size is strongly related to functional recovery [26], repeated rIPostC may be a simple and novel strategy to minimize the clinical impact of an ischemic stroke. Importantly, rIPostC is virtually cost-free, non-pharmacological, and non-invasive, and it can easily be added to the current treatment of stroke patients.

## Methods/design

### Aim

The aim of this trial is to examine the effect of repeated (twice daily) rIPostC during hospitalization on infarct size and clinical outcomes in ischemic stroke patients. In addition, we aim to explore the potential underlying (inflammatory) mechanisms for this effect of repeated rIPostC.

### Design

We will perform a randomized single-blind placebo-controlled clinical trial at the Radboud University Medical Centre (Radboudumc) in Nijmegen, the Netherlands. The study has been approved by the relevant ethical committee (CMO Arnhem-Nijmegen, registration number 2017–3711). This protocol has been registered with the Netherlands Trial Register (NTR6880).

### Patient population and randomization

Patients with an ischemic stroke who are being admitted to the emergency room of the Radboudumc are eligible for this study. Other inclusion criteria are: older than 18 years and a clinical diagnosis of an ischemic stroke (established by a neurologist, based on the World Health Organization criteria for stroke) [27]. Exclusion criteria include: unstable vital signs, admitted more than 12 hours after onset of symptoms, upper extremity injury, edema contra-indicating rIPostC, bilateral mastectomy, or axillary lymph node dissection. Patients with contra-indications for magnetic resonance imaging (MRI; e.g., those with a pacemaker, vascular clips, cochlear implants, or other implanted metal objects) will also be excluded because of the impossibility of assessing our primary outcome. All patients will provide written informed consent. For those patients who are not mentally capable of signing informed consent in the acute setting, oral assent will be obtained. This will allow the intervention to start while providing an opportunity for informed consent to be given when the patients or their caregivers are capable of making a well-considered decision. Patients who receive a change in diagnosis after inclusion will be excluded from the analysis of our primary outcome (infarct size).

After informed consent or oral assent has been given, randomization will be performed using a predefined table generated by the computer program SealedEnvelope. Patients will be randomized in a 1:1 ratio to rIPostC or sham conditioning by the coordinating investigator. Stratification will be performed for treatment received (thrombectomy, thrombolysis, or no revascularization treatment) to ensure equal allocation of these subgroups to rIPostC and sham conditioning.

### Intervention: repeated remote ischemic postconditioning

A manual blood pressure cuff will be placed around the upper arm after the diagnosis of an ischemic stroke. All participants will receive four cycles of 5-min inflation of the blood pressure cuff, followed by 5 minutes of reperfusion. This procedure will be performed twice daily (morning and afternoon) during the hospitalization after the ischemic stroke for a maximum of 4 days. The level of cuff inflation differs between the groups. Cuff inflation in the rIPostC-group is >20 mmHg above systolic blood pressure, mediating full blockage of the arterial inflow. Subjects in the control group receive cuff inflation to 50 mmHg, which will not induce any ischemia. The intervention will be administered by a trained researcher. Blood pressure will be recorded before the intervention.

### Primary outcome measure

The primary outcome measure will be the infarct size on day 4 after admission (or at discharge if discharge is before day 4), which will be compared between the intervention and control groups. The infarct size will be evaluated by diffusion-weighted MRI brain imaging using a 1.5-tesla MRI scanner (Siemens® Avanto). The infarct size will be annotated manually and analyzed on a dedicated work station by a trained researcher under the supervision of a neuroradiologist.

### Secondary outcome measures

(1) The validated and frequently used Modified Rankin Scale (mRS) [2, 28, 29] will be used to assess clinical outcomes after 12 weeks. To assess clinical outcomes at the end of hospitalization, the validated National Institutes of Health Stroke Scale [30] will be used. Both scores are calculated as standard after a stroke and will be assessed by a clinical physician or nurse from the neurology department.

After 12 months, we will also examine hospitalization, recurrent (cerebro)vascular events, and mortality. These data will be extracted from patient files at the Radboudumc.

(2) To assess quality of life, participants will be asked to fill out two questionnaires after 12 weeks and 1 year. The first questionnaire is the Stroke-Specific Quality of Life Scale (SSQoL), which is reliable and reproducible for self-reported quality of life outcomes in stroke patients [31]. The second questionnaire is the TOPICS-SF (The Older Persons and Informal Caregivers Survey Short Form), which is used for assessing patient-reported outcome measures. The TOPICS-SF has been shown to be a valid and feasible questionnaire for older patients [32, 33].

(3) Venous blood will be sampled during the first presentation at the emergency department and at the end of hospitalization to explore the impact of daily rIPostC on vascular, immune, and anti-inflammatory pathways. Whole blood will be spun down and serum will be stored at −80 °C for future analysis. For further analysis of possible underlying mechanisms, urine will also be sampled and stored at −80 °C at the end of hospitalization.

**Fig. 1:**
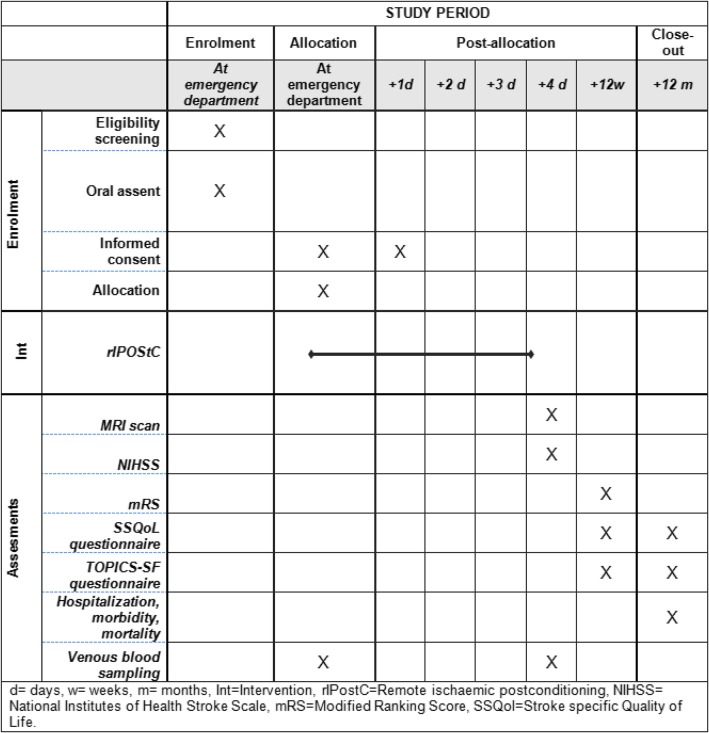
Schedule of enrolment, interventions, and assessments according to the SPIRIT guidelines [40]. d day, w week, m month, Int intervention, MRI magnetic resonance imaging, mRS Modified Rankin Scale, NIHSS National Institutes of Health Stroke Scale, rIPostC Remote ischemic postconditioning, SSQoL Stroke-Specific Quality of Life, TOPICS-SF The Older Persons and Informal Caregivers Survey Short Form

An overview of all outcomes and corresponding measurements is presented in Fig. 1.

### Sample size calculation

The sample size was estimated based on two trials [34, 35], which had a similar stroke population. Based on our clinical experience, we expect that a difference of 15 cm^3^ in infarct size between the two randomized groups should be considered as a clinically important difference. From the two trials, we estimated a standard deviation of 36 cm^3^. Based on α = 0.05 and power β = 0.80, we calculated that we need *n* = 90 per study arm. To account for an expected drop-out rate of 10%, we will include 100 patients in each group. This group size is comparable with other trials [20, 36] with the same intervention (rIPostC) and primary outcome (infarct size).

### Statistical analyses

Data will be analyzed according to an intention-to-treat analysis for all randomized patients. The primary outcome (infarct size) will be analyzed as a continuous variable using an independent *t*-test and presented with a 95% confidence interval for differences between both groups. Based on observations in previous research [2, 35, 37], we expect infarct size to be normally distributed. If the data are not normally distributed, a logarithmic transformation can be used. If this procedure fails, non-parametric alternatives will be used to analyze the data.

To analyze the mRS score, the odds ratio for a shift in the direction of a better outcome on the mRS will be assessed in both groups [38]. This ratio will be estimated with ordinal logistic regression and will be calculated for all possible cut-off values on the mRS.

For all continuous endpoints (e.g., quality of life, patient-reported outcomes, hospitalization, recurrent cerebrovascular accidents, and mortality, inflammatory, and vascular biomarkers), an independent *t*-test can be used to evaluate differences between usual care and rIPostC. We will consider the outcomes that are assessed at different time points (quality of life and patient-reported outcomes) as separate outcomes due to the possibility that there will be missing data after 12 months. If these continuous outcomes are, contrary to our expectations, not normally distributed, then a logarithmic transformation or non-parametric alternatives will be used.

Analyses will be done on the full data, and a sub-analysis will be performed excluding the patients who do not have a visible infarct on the MRI. Prior to analyses, the extent of missing data on all secondary outcomes will be evaluated and appropriate methods to handle missing data will be determined (e.g., multiple imputation). To ensure the analyses are blinded, all data will be blinded prior to analysis by an independent researcher. Full details of the analysis will be finalized in an analysis plan before the database is locked.

## Discussion

To our knowledge, this is one of the first clinical trials to investigate the effect of rIPostC after a stroke. Two previous studies have shown promising results for a single bout of RIC after a stroke [20, 21] and several other trials (registered under NCT02779712, NCT03045055, and NCT02189928 [36]) are currently being conducted that investigate the effect of a single bout of rIPostC in stroke patients. However, so far, no definitive conclusions on the effect of this intervention have been reported in this population. Our trial will study the effect of daily repeated rIPostC on both infarct size and clinical outcomes, but also aims to achieve a better understanding of the possible physiological mechanisms underlying this effect.

The primary outcome for this trial will be the difference in infarct size between the intervention and control groups. Infarct size in brain MRI scans was chosen because it can be assessed objectively. Moreover, infarct size has been used as a primary outcome in animal studies that showed a positive effect of rIPostC on infarct size [22, 39]. Diffusion-weighted imaging has been chosen to analyze the MRI data because it allows for a fast and reliable analysis of infarct size in the acute stage and does not require the use of contrast medium, thus minimizing patient burden.

We also aim to investigate clinical outcomes using the frequently used and validated mRS [2, 28, 29]. Importantly, this study has not been powered for this secondary outcome and, therefore, we may encounter challenges in detecting a significant difference between study arms. Nonetheless, infarct size has been reported to be a predictor for clinical outcomes in stroke patients [26] and the mRS scores will be used in the power calculation of a future clinical trial in which functional performance will be a clinical outcome.

Finally, we aim to include 200 patients. With approximately 250 stroke patients being admitted to the Radboudumc each year and with the eligibility criteria for this trial being very broad, we expect to recruit a sufficient number of patients during our recruitment period of ~2.5 years. We will include all stable adult stroke patients with a clinically diagnosed stroke who do not have contra-indications for an MRI or rIPostC and who are able to provide written informed consent. Based on this nonrestrictive participant selection, we expect no practical problems in recruiting a sufficient number of patients. An important advantage of our approach is that the results of our trial can be generalized to the larger population of stroke patients.

### Trial status

The first patient was randomized on 23 April 2018. At the time of the first submission of this manuscript, three patients had been recruited into the trial. Recruitment will continue until the complete sample size is achieved, which is expected to be in August 2020 at the latest.

## Additional file

Additional file 1: SPIRIT checklist for the REPOST trial. (DOCX 61 kb)

## Abbreviations

MRI: Magnetic resonance imaging
mRS: Modified Rankin Scale
NIHSS: National Institutes of Health Stroke Scale
Radboudumc: Radboud University Medical Centre
RIC: Remote ischemic conditioning
rIPostC: Remote ischemic postconditioning
SSQoL: Stroke-Specific Quality of Life
TOPICS-SF: The Older Persons and Informal Caregivers Survey Short Form

## References

[1] Roth GA (2015). Demographic and epidemiologic drivers of global cardiovascular mortality. N Engl J Med.

[2] Berkhemer OA, Majoie CB, Dippel DW (2015). Intraarterial treatment for acute ischemic stroke. N Engl J Med.

[3] Campbell BC (2015). Endovascular therapy for ischemic stroke with perfusion-imaging selection. N Engl J Med.

[4] Saver JL (2015). Stent-retriever thrombectomy after intravenous t-PA vs. t-PA alone in stroke. N Engl J Med.

[5] Emberson J (2014). Effect of treatment delay, age, and stroke severity on the effects of intravenous thrombolysis with alteplase for acute ischaemic stroke: a meta-analysis of individual patient data from randomised trials. Lancet.

[6] Nogueira RG (2018). Thrombectomy 6 to 24 Hours after Stroke with a Mismatch between Deficit and Infarct. N Engl J Med.

[7] Albers GW, et al. Thrombectomy for Stroke at 6 to 16 Hours with Selection by Perfusion Imaging. N Engl J Med. 2018;378:708-18. 10.1056/NEJMoa1713973 PMC6590673 29364767

[8] Bai J, Lyden PD (2015). Revisiting cerebral postischemic reperfusion injury: new insights in understanding reperfusion failure, hemorrhage, and edema. Int J Stroke.

[9] Chamorro A (2016). Neuroprotection in acute stroke: targeting excitotoxicity, oxidative and nitrosative stress, and inflammation. Lancet Neurol.

[10] Kharbanda RK (2002). Transient limb ischemia induces remote ischemic preconditioning in vivo. Circulation.

[11] Botker HE (2010). Remote ischaemic conditioning before hospital admission, as a complement to angioplasty, and effect on myocardial salvage in patients with acute myocardial infarction: a randomised trial. Lancet.

[12] Sloth AD (2014). Improved long-term clinical outcomes in patients with ST-elevation myocardial infarction undergoing remote ischaemic conditioning as an adjunct to primary percutaneous coronary intervention. Eur Heart J.

[13] Thijssen DH (2016). Repeated ischaemic preconditioning: a novel therapeutic intervention and potential underlying mechanisms. Exp Physiol.

[14] Murry CE, Jennings RB, Reimer KA (1986). Preconditioning with ischemia: a delay of lethal cell injury in ischemic myocardium. Circulation.

[15] Hausenloy DJ (2016). Ischaemic conditioning and targeting reperfusion injury: a 30 year voyage of discovery. Basic Res Cardiol.

[16] Iadecola C, Anrather J (2011). Stroke research at a crossroad: asking the brain for directions. Nat Neurosci.

[17] Wang Y (2015). Ischemic conditioning-induced endogenous brain protection: Applications pre-, per- or post-stroke. Exp Neurol.

[18] Hess DC (2015). Remote ischaemic conditioning-a new paradigm of self-protection in the brain. Nat Rev Neurol.

[19] Chen G, et al. Limb Remote Ischemic Postconditioning Reduces Ischemia-Reperfusion Injury by Inhibiting NADPH Oxidase Activation and MyD88-TRAF6-P38MAP-Kinase Pathway of Neutrophils. Int J Mol Sci. 2016;17(12):1971. 10.3390/ijms17121971 PMC5187771 27898007

[20] Hougaard KD (2014). Remote ischemic perconditioning as an adjunct therapy to thrombolysis in patients with acute ischemic stroke: a randomized trial. Stroke.

[21] England TJ (2017). RECAST (Remote Ischemic Conditioning After Stroke Trial): A Pilot Randomized Placebo Controlled Phase II Trial in Acute Ischemic Stroke. Stroke.

[22] Ren C (2015). Limb remote ischemic per-conditioning in combination with post-conditioning reduces brain damage and promotes neuroglobin expression in the rat brain after ischemic stroke. Restor Neurol Neurosci.

[23] Doeppner TR (2018). Very Delayed Remote Ischemic Post-conditioning Induces Sustained Neurological Recovery by Mechanisms Involving Enhanced Angioneurogenesis and Peripheral Immunosuppression Reversal. Front Cell Neurosci.

[24] Meng R (2012). Upper limb ischemic preconditioning prevents recurrent stroke in intracranial arterial stenosis. Neurology.

[25] Meng R (2015). Ischemic Conditioning Is Safe and Effective for Octo- and Nonagenarians in Stroke Prevention and Treatment. Neurotherapeutics.

[26] Zaidi SF (2012). Final infarct volume is a stronger predictor of outcome than recanalization in patients with proximal middle cerebral artery occlusion treated with endovascular therapy. Stroke.

[27] Hatano S (1976). Experience from a multicentre stroke register: a preliminary report. Bull World Health Organ.

[28] Middleton S (2011). Implementation of evidence-based treatment protocols to manage fever, hyperglycaemia, and swallowing dysfunction in acute stroke (QASC): a cluster randomised controlled trial. Lancet.

[29] Quinn TJ (2009). Functional outcome measures in contemporary stroke trials. Int J Stroke.

[30] Abdul-Rahim AH (2015). National Institutes of Health Stroke Scale Item Profiles as Predictor of Patient Outcome: External Validation on Safe Implementation of Thrombolysis in Stroke-Monitoring Study Data. Stroke.

[31] Muus I, Williams LS, Ringsberg KC (2007). Validation of the Stroke Specific Quality of Life Scale (SS-QOL): test of reliability and validity of the Danish version (SS-QOL-DK). Clin Rehabil.

[32] Hems M (2017). Patient reported outcome measures in geriatric care: first experiences. Tijdschr Gerontol Geriatr.

[33] Lutomski JE (2017). Responsiveness of the full-length and short form of The Older Persons and Informal Caregivers Survey. J Am Med Dir Assoc.

[34] Rosso C (2011). Hyperglycemia and the fate of apparent diffusion coefficient-defined ischemic penumbra. AJNR Am J Neuroradiol.

[35] Rosso C (2012). Intensive versus subcutaneous insulin in patients with hyperacute stroke: results from the randomized INSULINFARCT trial. Stroke.

[36] Pico F (2016). A multicenter, randomized trial on neuroprotection with remote ischemic per-conditioning during acute ischemic stroke: the REmote iSchemic Conditioning in acUtE BRAin INfarction study protocol. Int J Stroke.

[37] Schiemanck SK (2006). Predictive value of ischemic lesion volume assessed with magnetic resonance imaging for neurological deficits and functional outcome poststroke: A critical review of the literature. Neurorehabil Neural Repair.

[38] Saver JL (2007). Novel end point analytic techniques and interpreting shifts across the entire range of outcome scales in acute stroke trials. Stroke.

[39] Hahn CD (2011). Remote ischemic per-conditioning: a novel therapy for acute stroke?. Stroke.

[40] Chan AW (2013). SPIRIT 2013 explanation and elaboration: guidance for protocols of clinical trials. BMJ.

